# Fine Mapping of a QTL Associated with Kernel Row Number on Chromosome 1 of Maize

**DOI:** 10.1371/journal.pone.0150276

**Published:** 2016-03-01

**Authors:** Claudia I. Calderón, Brian S. Yandell, John F. Doebley

**Affiliations:** 1 Laboratory of Genetics, University of Wisconsin-Madison, Madison, Wisconsin, United States of America; 2 Department of Statistics and Department of Horticulture, University of Wisconsin-Madison, Madison, Wisconsin, United States of America; Universidad Miguel Hernández de Elche, SPAIN

## Abstract

The genetic factors underlying changes in ear morphology, and particularly the inheritance of kernel row number (KRN), have been broadly investigated in diverse mapping populations in maize (*Zea mays* L.). In this study, we mapped a region on the long arm of chromosome 1 containing a QTL for KRN. This work was performed using a set of recombinant chromosome nearly isogenic lines (RCNILs) derived from a BC_2_S_3_ population produced using the inbred maize line W22 and teosinte (*Zea mays ssp*. *parviglumis*) as the parents. A set of 48 RCNILs was evaluated in the field during the summer of 2013 in order to perform the mapping. A QTL for KRN was found that explained approximately 51% of the phenotypic variance and had a 1.5-LOD confidence interval of 203 kb. Seven genes are described in this interval. One of these candidate genes may have been the target of domestication processes in maize and contributed to the shift from two kernel row ears in teosinte to a highly polystichous ear in maize.

## Introduction

Since the beginning of the domestication process of maize, one of the major selective pressures that humans placed upon the maize ancestor teosinte was a selection for an increase in the size of the female inflorescence, or ear. This morphological modification of the ear involved an increase in ear length, an increase in ear diameter, and an increase in the number and size of the kernels. Due to the importance of maize as a staple grain crop in the world, researchers have been drawn to the challenge of understanding the genetic basis of floral architecture in maize, with the long term goal of increasing the number of seeds per inflorescence and enhancing crop yields. Towards this end, scientists have attempted quantitative trait loci (QTL) mapping as a tool for examining the genetic architecture of traits of interest.

A number of specific loci have been identified that affect maize morphological evolution. Large-effect QTLs such as *teosinte branched1 (tb1)*, *teosinte glume architecture1 (tga1*), and prolificacy (*gt1*) have been successfully fine-mapped and have revealed key genetic changes that occurred during the domestication of maize [1–3]. The *tb1* gene is a member of the TCP family of transcriptional regulators and controls differences in plant architecture, particularly the increase in apical dominance in maize relative to teosinte [4]. The allele of *tga1* present in teosinte results in the formation of kernels that are encased in a hardened fruitcase. By contrast, the *tga1* allele present in maize alters the development of the fruitcase, leaving the kernel exposed in the ear and more accessible for harvest [5]. *gt1* is a locus found on the short arm of chromosome 1 that affects prolificacy (the number of ears on a plant). *tb1*, *tga1* and *gt1* all encode transcriptional regulators [1, 3]. Genes encoding transcriptional regulators that control a major domestication trait have also been identified in other grains, such as the Q gene in wheat [6], *Shattering1* (*Sh1*) in sorghum [7], and the rice genes *Shattering4* (*sh4*), *Shattering1* (*SH1*), *Shattering abortion1* (*SHAT1*), and *red coloration of the pericarp* (*Rc*) [2, 6–9].

In contrast to traits that have been shown to be largely controlled by single genes of large-effect [1, 2], several other domestication-related traits appear to be controlled by multiple genes of small effect and have proven harder to dissect. Inheritance of kernel row number (KRN) is polygenic, and a number of QTLs throughout the maize genome have been previously described for that trait [10–27]. In a prior study, twenty-five QTLs for KRN were found using a BC_2_S_3_ mapping population [12]. The QTL with the highest LOD score for KRN was found on chromosome 5 (LOD 65.83), however a recent study determined that this KRN QTL fractionated into multiple linked QTL on this chromosome, suggesting a complex form of inheritance [28]. The QTL with the second highest LOD score for KRN in this population was found on the long arm of chromosome 1 (LOD of 56.91) and will be referred to here as KRN1.4.

In this study, our focus was the identification of the causal gene underlying KRN1.4. First, we tested two heterogeneous inbred families (HIFs) segregating for KRN1.4 to confirm the general position and effect of KRN1.4. Then, we isolated 48 recombinant chromosome nearly isogenic lines (RCNILs) from the HIFs for fine-mapping this QTL. The RCNILs did not segregate into two phenotypic classes for kernel row number corresponding to the maize and teosinte haplotypes. Rather, they displayed a continuous range of trait values such that fine-mapping the QTL to a single gene was not possible. While we could not explicitly determine which gene underlies this QTL, the RCNILs enabled us to define a narrower 1.5-LOD confidence interval for the KRN1.4 within which there are just seven genes. We discuss which of these seven genes may be more likely to contribute to KRN variation.

## Materials and Methods

### Mapping population

For our study, we studied two heterogeneous inbred families (HIFs) derived from a set of BC_2_S_3_ maize-teosinte recombinant inbred lines (RILs) previously generated by our team for use in QTL mapping studies [12]. These BC_2_S_3_ lines were derived from an initial cross between a Midwest maize inbred (W22) and an accession of Balsas teosinte, *Zea mays* ssp. *parviglumis* (CIMMYT 8759) [12]. This subspecies of teosinte has been reported to be the closest wild relative of maize [29]. W22 was used as the recurrent parent to produce advanced backcross lines with a largely W22 genetic background. The two HIFs we used for our study, MR0194 and MR0579, segregate for a teosinte introgression in the 1.5-LOD support interval of KRN1.4 [12]. The previously defined coordinates of the borders of KRN1.4 were 292,880,151 bp and 293,153,020 bp, based on the B73 RefGen_v2 assembly [30, 31].

We grew 1,994 individual plants, 915 of MR0194 and 1079 of MR0579 during the summer of 2012 at the West Madison Agricultural Research Station (Verona, WI). To identify recombinant chromosomes, we genotyped the plants using two markers that flank KRN1.4, umc1737 and cic001 (S1 Table). Thirty-one plants with a cross-over between the two markers were obtained from MR0194 and 17 such plants from MR0579. These 48 recombinant chromosome plants were then self-pollinated. Ears were subsequently harvested, phenotyped, and processed to save seed. Seeds harvested from these individuals were then grown in the greenhouse (Fall 2012), genotyped, and self-pollinated to produce individuals homozygous for the recombinant chromosomes, resulting in a total of 48 unique RCNILs. Duplicates of the RCNIL stocks were included if available. In addition, eight lines homozygous for maize DNA in the KRN1.4 interval and twelve lines homozygous for teosinte DNA in the KRN1.4 interval were also included in the study as controls (S2 Table).

### Molecular markers and genotyping

Genomic DNA from young leaf tissue of each RCNIL was extracted using a standard CTAB method [32]. Eighteen PCR-based markers were developed targeting single nucleotide polymorphisms or indels (S1 Table). Genotyping was done via Sanger sequencing or by capillary electrophoresis. Amplicons were sequenced in both directions, cleaned using CleanSEQ magnetic beads (Agencourt, Beverly, MA, USA), and analyzed by the Biotechnology Center Sequencing Facility at the University of Wisconsin-Madison using an ABI3730 DNA sequencer (Applied Biosystems, Foster city, California, USA). The sequences were edited and aligned using Sequencher v.5.1 (GeneCodes, Ann Arbor, Michigan, USA) to manually score polymorphisms. For capillary electrophoresis, fluorescently labeled primers (FAM or HEX) were used for PCR amplification to label fragments that were subsequently mixed with Geneflo 625 DNA ladder labeled with ROX (Chimerx, Milwaukee, WI, USA) as a size standard. DNA fragments and standards were denatured with Hi-Di ^™^ Formamide (Applied Biosystems, Foster city, CA, USA) prior to loading on the ABI37300 analyzer. Data was visualized using the Genescan software package from ABI.

### Phenotyping

KRN was obtained by counting the number of kernels around the diameter of the middle part of the top ear. The plants were grown at the West Madison Research Station (Verona, WI). The spacing between adjacent plots was 30 inches, with two foot walkways separating the end and start of each plot. The two HIFs were phenotyped in 2012 including those plants that had recombinant chromosomes as well as control plants that were either all maize, all teosinte or heterozygous at the marker loci. Of the plants evaluated, 226 plants had the homozygous maize genotype, 207 the homozygous teosinte genotype, 222 were heterozygous, and 199 were recombinant. The plots were 7.6 meters long. For the RCNILs, plants were grown in a randomized complete block design with four blocks, 119 plots per block, and 16 plants per plot. The plots were 4.5 meters long. Up to 14 plants were phenotyped per plot.

### Statistical analysis

Basic statistical analysis of the data was done using R [33] and SAS software, version [9.1] (SAS Institute Inc., Cary, NC). An analysis of variance (ANOVA) was used to test differences in trait means among phenotypic classes in the two initial HIFs. The least squared means (LSM) of KRN for the RCNILs were computed using the restricted maximum likelihood estimation (REML) using the MIXED procedure of SAS and the following model:

y = μ + αi (k) + βj + δk + (αβ)ij(k) +ε(ijk)l

where **y** represents the phenotypic mean value for each trait, *μ* is the overall mean, *α* is the effect of the i^th^ RCNIL line (genetic stock) nested within family, *β* is the effect of j^th^ block, *δ* is the effect of the k^th^ family, (*αβ*) is the ij^th^ block * RCNIL nested in family interaction and *ε* represents residual error. *β* and *ε* are independent random variables. LSM extracted from this analysis were used as the trait values for QTL mapping analyses [34].

We calculated broad-sense heritability (*H*^2^) on a plot mean basis as

$$H^{2}=\;\frac{\sigma_{g}^{2}}{\sigma_{g}^{2}+\sigma^{2}},$$

where *σ*^2^_g_ is the genetic variance and *σ*^2^ is the error variance. We used the MIXED procedure of SAS to fit a linear random-effect model for the estimation of the variance components [34].

### Fine mapping KRN1.4

To assess whether the RCNILs would segregate into two phenotypic classes, or “Mendelize”, we used column graphs with the LSM (±SE) arranged in order from high to low and aligned with a map of the teosinte:maize chromosomal segments in the QTL interval.

### QTL mapping

We performed QTL mapping with the genotype and trait data from the RCNILs to more precisely map KRN1.4. Eighteen PCR-based markers were used to genotype the region of interest for each of the RCNILs and control lines, and QTL analysis was performed using R/qtl [3, 12, 20, 28, 35–37]. We used *fill*.*geno* to fill in the missing genotypes (0.86%) with a single imputation based on flanking markers. A QTL linear model was fitted using the Haley-Knott regression method as implemented by the R/qtl command *scanone* as an approximation to standard interval mapping and with an assumed genotyping error rate of 0.01[35, 38]. We verified that with 0.86% missing marker values, the method of handling missing data (marker regression or Haley-Knott regression) had negligible effect on the results. Statistical significance of the peak LOD score was assessed using 10,000 permutations of the data and a 5% significance level threshold. We also calculated the 1.5-LOD support intervals for the location of the identified QTL. HIF membership of each RCNIL was included as a covariate in the linear model. In order to assess the support of individual terms in the QTL model, we specified the drop-one-term analysis from *fitqtl* which compares the fit of the full model with all the terms to a reduced model in which terms are omitted. The model that showed an improvement in fit was retained. The *fitqtl* command in R/qtl was also used to estimate the QTL effect.

## Results

### RCNIL generation

Of the initial 1,994 plants from the two founding HIFs, we collected genotypic data for 1,903 individuals (874 from MR0194 & 1,029 from MR0579) in order to identify recombinant chromosomes in the summer of 2012. We also performed a preliminary phenotypic analysis and ANOVA of 700 individuals to check the assumption that a QTL affecting KRN was segregating in this population. In this analysis, 226 of the plants were homozygous for the maize genotype at the two markers flanking the KRN1.4 interval, 207 were homozygous for the teosinte genotype, and 222 were heterozygous. This analysis was performed to test the null hypothesis of equal means among the three genotypic classes. A statistically significant difference in KRN means (F-test, p < 0.001) was obtained, indicating an effect of genotype in this interval on the KRN phenotype (Table 1). Also, to estimate the degree of dominance and additivity, we compared the trait values of maize, teosinte and heterozygous genotypic classes at KRN1.4. The additive effect was 0.72 kernel rows. The dominance/additivity ratio obtained was 0.2, indicating additive gene action (Table 2).

**Table 1:** Analysis of Variance of KRN.

	Df		F value		Pr (>F)	
	MR0194	MR0579	MR0194	MR0579	MR0194	MR0579
**Genotypes**	2	2	29.348	45.219	2.2e-12***	2.2e-16***
**Residuals**	305	297				

Df, degrees of freedom. Analysis of variance is shown for both of the heterogeneous inbred families: MR0194 and MR0579.

*** Significance at the 0.001 level.

**Table 2:** Gene action for KRN.

	Genotypic classes			
Trait	M/M	H/H	T/T	N
kernel row number	13.9557522	13.378378438	12.5120773	655

Genotypic classes obtained by the two markers flanking the KRN1.4 interval. M/M indicates homozygous for maize DNA at the two markers, H/H indicates heterozygous, and T/T indicates homozygous for teosinte.

From those 1,903 individuals screened in the summer of 2012, 48 plants with a cross-over between the two markers that flank the QTL interval were obtained. These plants were then self-pollinated to generate RCNILs. Two types of control lines (8 homozygous maize and 12 homozygous teosinte) for the QTL interval were also selfed at this time. In total, 68 homozygous RCNILs were obtained.

**Fig 1:**
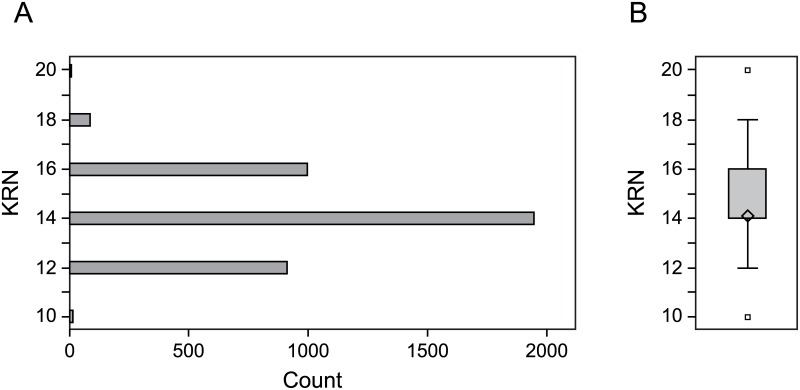
Phenotypic Distribution of KRN. (A) The total number of individuals with a given KRN value are indicated on the x-axis. Only the top ear from each plant was scored. (B) Boxplot representation of the data shown in (A).

The RCNILs were grown in a field trial in 2013 consisting of four replicates, with 119 plots per block and 16 plants per plot. Least squared means for KRN were calculated from these data. The phenotypic distribution of the KRN trait among the RCNILs is normal as shown in Fig 1 and S1 Fig. We also used the RCNILs to estimate broad-sense heritability for KRN from the variance components obtained from SAS. The heritability for KRN was 0.95.

### Attempted fine-mapping of a QTL for KRN

**Fig 2:**
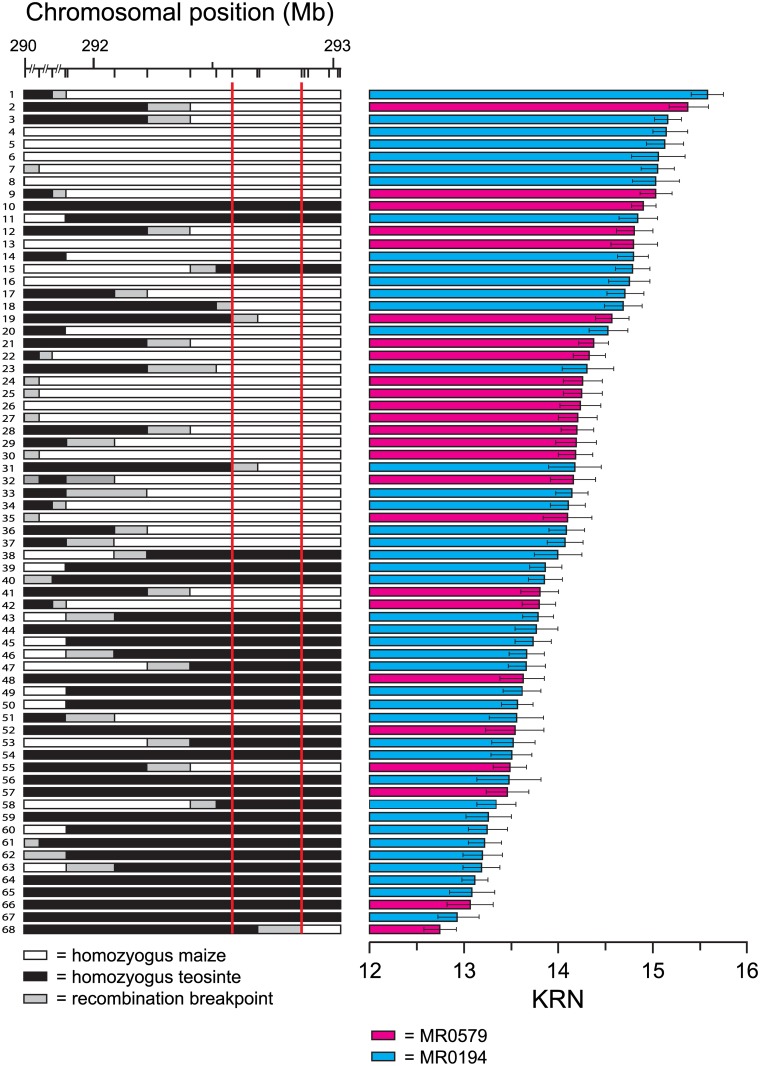
RCNILs sorted by KRN phenotype. Left panel presents the genetic maps of 68 RCNILs for the interval on the long arm of chromosome 1 containing KRN1.4. It shows the physical positions of the 18 genetic markers used in this study (S1 Table) based on the RefGen_v2 assembly. White bars indicate DNA homozygous for the maize W22 allele, black bars DNA homozygous for the teosinte allele, and gray boxes the regions within which the recombination break points are located. Least squared means values for KRN with standard error bars for each line are shown in the right panel. Blue bars represent KRN of RCNILs derived from the MR0194 population and pink bars KRN of RCNILS derived from the MR0579 population. Red lines delineate the interval with the best fit of genotype and phenotype.

Fine-mapping of a gene controlling a trait of interest is possible if the RCNILs segregate into two classes with respect with that trait, i.e. the trait “Mendelizes”. In the case of KRN1.4, however, we determined that the KRN phenotype did not behave in a Mendelian fashion in our RCNILs (Fig 2). Nevertheless, visual inspection of Fig 2 reveals a pattern in which a large majority of the lines with the highest KRN values have the maize genotype in the interval between markers cic035 and cic011. However, the correspondence between genotype and phenotype in this interval is far from perfect. Because of this situation, fine mapping the causative mutation to a defined chromosomal position was not possible, so we performed a QTL analysis to further narrow down the genomic region controlling the KRN phenotype.

### QTL mapping

**Fig 3:**
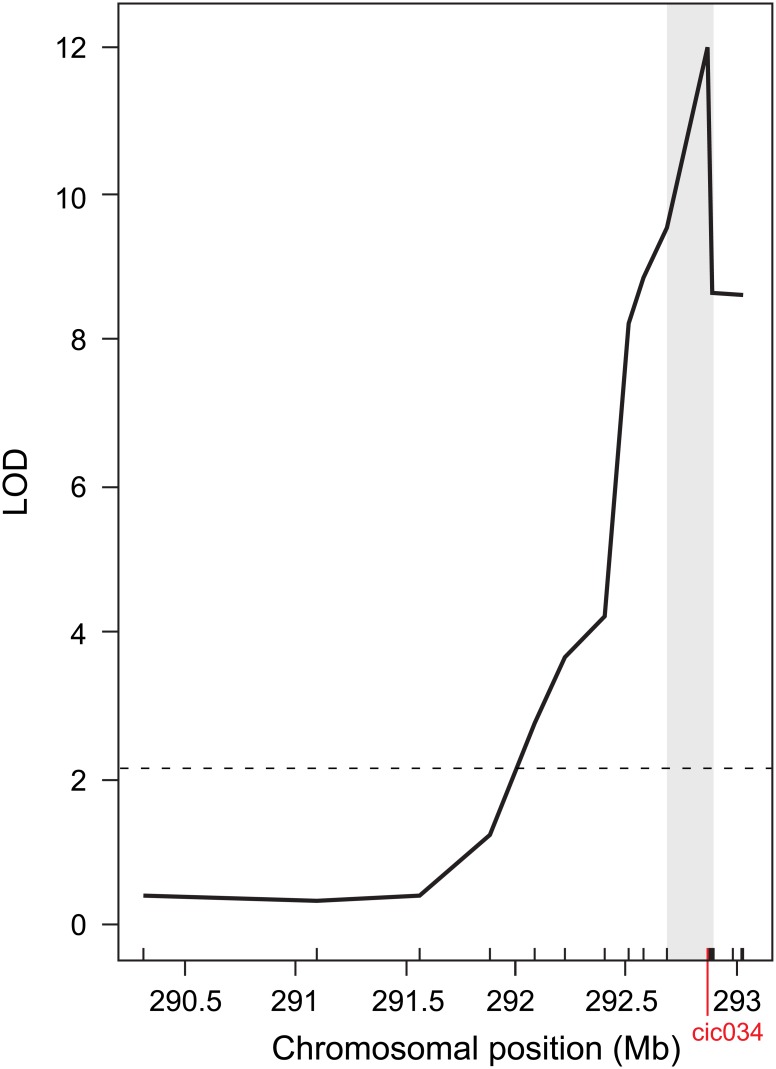
QTL Map of KRN1.4. Molecular markers used in this study are indicated by vertical lines above the x-axis. Marker coordinates are given in S1 Table. Chromosomal positions in megabases are based on B73 RefGen_v2. Dashed line indicates the 5% significance threshold level. Vertical gray bar indicates the 1.5-LOD confidence interval.

QTL Mapping of the KRN1.4 region using a collection of 18 polymorphic markers was conducted using 48 RCNILs obtained from the original 1,903 plants screened in 2012. A QTL for KRN was detected on chromosome 1 (Fig 3). The *fitqtl* analysis suggests strong evidence for a significant family (HIF) effect (p = 2.6x10^-6^), but no evidence for differential QTL effects by family (*p* = 0.725). The highest peak of the QTL was found at marker cic034 with an LOD score of 11.94, which strongly exceeds the 5% significance threshold level of 2.15. The cic034 marker corresponds to position 292,878,833 bp on the B73 RefGen_v2 assembly (S1 Table, Fig 3).

**Fig 4:**
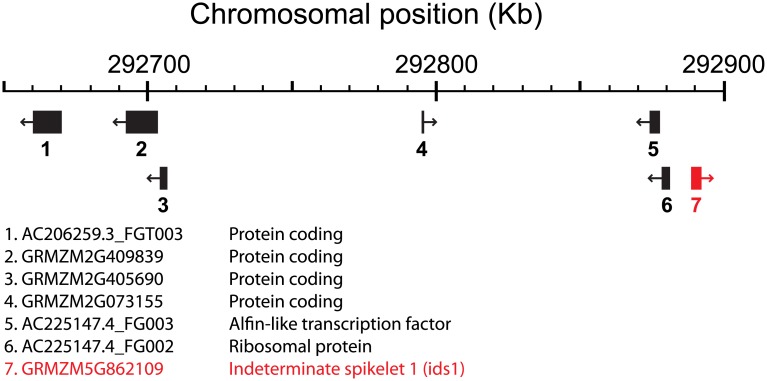
Genes in the 1.5-LOD confidence interval for KRN1.4. Black boxes represent genes described in the 1.5-LOD support interval for KRN1.4. The red box indicates the position of the *indeterminate spikelet 1* (*ids1*) gene. Arrows indicate transcriptional direction.

The 1.5-LOD interval of the QTL for KRN spanned 203 kb, from position 292,686,855 bp to 292,890,107 bp of the physical map (B73 RefGen_v2). This interval contains seven genes (Fig 4). It should be noted that the right hand boundary of the KRN1.4 interval was established in our study based on a evidence from a single recombinant line, thus it is not strongly supported at this time. We also found that KRN1.4 had an additive effect of +1.0561 where the maize allele conditions an increase of KRN, which is slightly larger than the +0.71 value found in the HIFs. In the model, KRN1.4 explained 50.48% of the variance.

## Discussion

### The genetic architecture of kernel row number

The BC_2_S_3_ population used in this study was derived from a cross between maize and its progenitor teosinte. This population has provided a useful system for studying QTLs underlying the domestication process in maize [12]. The parental lines used to create this mapping population were chosen because they differed dramatically in ear morphology and plant architecture. While teosinte (*Zea mays ssp*. *parviglumis*) produces ears with only two interleaved rows of about 8–12 kernels, the maize ear (*Zea mays*) can have 20 rows or more per ear.

The process of fine-mapping a QTL involves isolating a large number of recombinant chromosomes in the region of interest from a segregating population. Fine-mapping has been successfully used to narrow down a number of QTLs affecting domestication traits to single gene of large effect, including *tga1* [5], *tb1* [39], *prol1*.*1* [3], *ZmCTT* [40], and *vgt1* [41]. However, fine-mapping does not always succeed as in our case where the RCNILs fail to segregate into two phenotypic classes.

Because our RCNILs did not segregate in a Mendelian fashion for the least squared means of KRN, we could not explicitly determine which gene(s) underlies this trait. Potential explanations for this situation include the presence of additional loci in the genome that affect KRN and/or environmental factors affecting the phenotype. The heritability of KRN in our study was high, with a value of 0.95. In other published studies on KRN, this trait also showed a high heritability [42]. Given high heritability and minimal environmental effects, environmental variance can not explain the failure of KRN for the RCNILs to Mendelize in our study. Thus, the differences among the RCNILs is most likely influenced by genetic factors elsewhere in the genome outside of the QTL interval for KRN1.4. Since the RCNILs were derived from BC_2_S_3_ plants, they contain multiple teosinte segments around the genome. For the BC_2_S_3_ population, KRN was a highly polygenic traits with a minimum of 25 QTL detected [12]. Thus, the expectation is that the RCNILs differ for other QTL in addition to KRN1.4.

Because fine-mapping failed, we performed a QTL analysis in R/qtl. The QTL interval in which we mapped KRN1.4 contains seven genes from the maize Filtered Gene Set (FGS) (Table 3, Fig 4). Genes 1 to 4 (Table 3, Fig 4) code for proteins whose function could not be inferred from orthology to genes described in other species. Located under the peak of the QTL is gene 5 (AC225147.4_FG003), an alfin-like transcription factor shown to be involved in chromatin-mediated transcriptional regulation [43]. It is a PHD zinc finger protein that has the potential to bind to *cis*-acting elements in the promoter regions of target genes [44]. The highly conserved nature of the PHD finger motif suggests that alfin-like proteins may have relevant biological roles in plants [45]. Gene 6 (AC225147.4_FG002) is a small-subunit ribosomal gene [30, 31]. Finally, another interesting candidate that fell within the 1.5-LOD support interval is gene 7, *Indeterminate spikelet1* (*ids1*). *Ids1* is a gene that encodes an APETALA2-like transcription factor that plays a role in the ABC model of flower development [46, 47]. In maize, the function of *ids1* is to regulate inflorescence branching, floral meristem determinacy, and spikelet meristem determinacy. *Ids1* would seem a strong candidate for KRN1.4 given its role in regulating inflorescence branching. Since mutant alleles of *ids1* are available in maize, one strategy for testing the hypothesis that KRN1.4 is caused by a mutation in the *IDS1* gene would be to cross one of our RC-NIL lines showing the teosinte KRN phenotype with a maize *ids1* line. Analysis of the F1 plants could reveal if KRN1.4 is indeed caused by a mutation in *IDS1*. This candidate gene is particularly interesting because ids1 mutants have been previously shown to have a reduced kernel row number [48].

**Table 3:** Genes from the Maize Filtered Gene Set in the 1.5 LOD Confidence Interval of KRN1.4.

No.	Gene models	B73 RefGen_v2	Functional Characterization ^a^
1	AC206259.3_FGT003	chr1:292,660,134–292,671,866	Protein coding
2	GRMZM2G409839	chr1:292,694,407–292,704,781	Protein coding
3	GRMZM2G405690	chr1:292,705,562–292,709,078	Protein coding
4	GRMZM2G073155	chr1:292,796,431–292,797,030	Protein coding
5	AC225147.4_FG003	chr1:292,875,307–292,879,433	Alfin-like transcription factor 15; PHD finger protein
6	AC225147.4_FG002	chr1:292,879,723–292,882,219	Ribosomal protein S23 family protein
7	GRMZM5G862109	chr1:292,889,740–292,893,983	*Indeterminate spikelet 1* (*ids1*), *tasselseed 6* (*ts6*)

B73 RefGen_v2: B73 reference genome sequence assembly version 2.

^a^ Information retrieved from the Maize Genetics and Genomics Database, MaizeGDB [30, 31].

It should be noted that the candidate gene list described above was obtained from the maize genome annotation. It is possible that the teosinte genome contains a different complement of genes in this interval, but since a fully annotated teosinte genome is not yet available, the maize genome currently provides us with the best resource for predicting gene content in this region. When an annotated teosinte genome becomes available it should be possible to reevaluate this interval for additional candidate loci.

To further narrow down the best candidate among these seven genes, we considered the results of a published genomic scan for selection that identified loci potentially involved in domestication and improvement [49]. None of our seven genes appeared in the lists of selected genes. Next, we examined a published study of gene expression in maize and found that six of the seven genes in our interval were expressed in the ear [50]. The only gene whose expression was not detected in the ear is GRMZM2G073155 (Table 3, S3 Table).

Among the six genes with expression in the ear, three show some evidence of *cis*-regulatory difference between maize and teosinte [50]. One of these (GRMZM2G409839 –unknown function) shows higher expression in maize than in teosinte due to *cis* effects alone. Another (AC225147.4_FG002 –ribosomal protein) shows higher expression in maize than in teosinte due to a combination of *cis* and *trans* effects. The third, which is *ids1*, has a more complicated regulation showing higher expression of the maize allele in maize-teosinte F1 hybrids, but lower expression of the maize allele when pure maize is contrasted to pure teosinte. [50]. There is not straightforward biological interpretation of this regulatory pattern in regards to the hypothesis that *ids1* is the gene that underlies KRN1.4, however the high expression of the maize allele in F1 hybrids argues that the maize and teosinte alleles have *cis* regulatory differences. The reverse expression pattern in the pure parents could be an artifact due to the difficulty of obtaining equivalent developmental states of immature maize and teosinte ears.

The next step in this project will be to map the KRN1.4 QTL to a single gene. In order to achieve this goal, the following steps could be taken. First of all, identifying more recombinant individuals could help resolve even further the definition of the interval containing KRN1.4. Also, it seems likely that variation in other regions segregating in the RCNILs could contain genes affecting KRN. To account for these genes, future analyses could incorporate genotyping with genome-wide markers to capture other regions of the genome that may be affecting KRN in addition to our interval on chromosome 1.

This work has refined our understanding of the location of a QTL on chromosome 1 that affects KRN. We have narrowed down the location of this QTL to a region containing seven genes from the Maize Filtered Gene Set. It is intriguing that two transcription factors are included in this interval, including the *ids1* gene with an established role in meristem function and inflorescence branching. Until the causative mutation has been identified, however, all of the candidate genes in this list must be considered as possibilities, as well as other functional elements that may not be currently annotated within this interval. Given the short list of genes in this region, it should now be practical to pursue candidate-gene based approaches to determine the causative mutation as well as potential changes in the timing or level of gene expression [51] that could be responsible for the KRN1.4 QTL. Ultimately, that information should provide new insights into the molecular mechanisms underlying the domestication process in maize.

## Supporting Information

S1 Fig Histogram of the Least Squared Means of Kernel Row Number. (DOCX)

S1 Table Markers used for QTL mapping. (DOCX)

S2 Table Genetic stock lines used for phenotyping. (DOCX)

S3 Table Expression Data of the Genes located within the 1.5 LOD Confidence Interval of KRN1.4. (DOCX)
