# Supplementary material for: Fine Mapping of a QTL Associated with Kernel Row Number on Chromosome 1 of Maize
Source: PLoS One. 2016 Mar 1;11(3):e0150276. doi: 10.1371/journal.pone.0150276 (PMC4773258; doi:10.1371/journal.pone.0150276)
Supplement: S1 Fig — (DOCX) [file pone.0150276.s001.docx]

**S1 Fig. Histogram of the Least Squared Means of Kernel Row Number**

Least squared means of Kernel row number for each of the RCNILs planted in the field in 2013.
