# Supplementary material for: Fine Mapping of a QTL Associated with Kernel Row Number on Chromosome 1 of Maize
Source: PLoS One. 2016 Mar 1;11(3):e0150276. doi: 10.1371/journal.pone.0150276 (PMC4773258; doi:10.1371/journal.pone.0150276)
Supplement: S1 Table — (DOCX) [file pone.0150276.s002.docx]

**S1 Table. Markers used for QTL mapping.**

| **Marker** | **B73 RefGen_v2** | **Forward primer** | **Reverse primer** |
| --- | --- | --- | --- |
| **umc1737** | 290,307,743 | ATGCTTCTCTTCAGAAGCCATCC | TAGCTAGGTAGTGATGTGCGTGCT |
| **cic020** | 291,101,573 | GACCAACAAGGCACCTTATGA | TGGGGAGCAGCACAGTAGTAG |
| **cic021** | 291,559,995 | TCATCAACTCCGACAAGATCC | ACCATGCTGCTGCTGTACTCT |
| **cic038** | 291,888,375 | CAGAACGCCACTGATGGTTAT | TTCGAACATCAACCTTTCCAC |
| **cic028** | 292,090,089 | ACCTGCGTTTGCAAAATACAC | CTAGATTGGGAGCGAGAAACC |
| **cic029** | 292,225,982 | CGTTGCGAAATCTCTCATCTC | GGACAACCGAGGCATTAACAC |
| **cic023** | 292,401,688 | TTTTGATGCTGTCCCACCTAC | TGGGAGCTGTGAACCATAGAC |
| **cic033** | 292,517,460 | GACATCGTGGAGATGAAGTCG | GAGAACAAGAGCATCGACAGC |
| **cic025** | 292,576,105 | GCACCATGAGTTAAAGCAAGC | CGGTTAAAATGTGTTCGGTGT |
| **cic027** | 292,686,855 | CCCATTCCAGGGATATAGTGG | CTCATGGTTTGAGTGTTTCAGC |
| **cic034** | 292,878,833 | GAATCCCAACTCCTGTGTGTG | GCGTGTGCTACAGGACAGAAT |
| **cic035** | 292,880,176 | AGAAACCCTTTGCTGGATCAT | GAGGTCCCAGAACTCCAAAAG |
| **cic011** | 292,890,107 | GATGGTGCTGGATCTCAATGT | ATCACCAGTCCACAACGAAAC |
| **cic015** | 292,890,892 | CGGTTAGTTTTACTGCGCTTG | ATCACCAGTCCACAACGAAAC |
| **cic001** | 292,893,690 | CGCAGTTCCCGACTACTACAA | AAATTGGCCTTTTGCTTCAGT |
| **cic016** | 292,990,224 | GCCTTTAGCCCTTTACTATGCAA | CTCAGCTGTGCTTTTCAGCAG |

B73 RefGen_v2 column gives the position of the marker in (bp) on the chromosome 1 physical map of B73 reference genome assembly version 2.
