# Supplementary material for: Fine Mapping of a QTL Associated with Kernel Row Number on Chromosome 1 of Maize
Source: PLoS One. 2016 Mar 1;11(3):e0150276. doi: 10.1371/journal.pone.0150276 (PMC4773258; doi:10.1371/journal.pone.0150276)
Supplement: S2 Table — (DOCX) [file pone.0150276.s003.docx]

**S2 Table. Genetic stock lines used for phenotyping**

| **No.** | **Stock** | **Seed lot** |
| --- | --- | --- |
| 1 | W22-KRN1L_IN0803 RCL | 0803_27.09 |
| 2 | W22-KRN1L_IN1761 RCL | 1761_51.01 |
| 3 | W22-KRN1L_IN0911 RCL | 0911_33.07 |
| 4 | W22-KRN1L_IN0248 MCL | 0248_05.03 |
| 5 | W22-KRN1L_IN0088 MCL | 0088_35.01 |
| 6 | W22-KRN1L_IN0680 MCL | 0680_21.13 |
| 7 | W22-KRN1L_IN0275 RCL | 0275_07.03 |
| 8 | W22-KRN1L_IN0610 MCL | 0610_17.05 |
| 9 | W22-KRN1L_IN1643 RCL | 1643_49.10 |
| 10 | W22-KRN1L_IN0904 TCL | 0904_32.21 |
| 11 | W22-KRN1L_IN0809 RCL | 0809_28.08 |
| 12 | W22-KRN1L_IN0918 RCL | 0918_36.10 |
| 13 | W22-KRN1L_IN0963 MCL | 0963_59.05 |
| 14 | W22-KRN1L_IN0719 RCL | 0719_24.01 |
| 15 | W22-KRN1L_IN0687 RCL | 0687_22.03 |
| 16 | W22-KRN1L_IN0257 MCL | 0257_06.07 |
| 17 | W22-KRN1L_IN0248 RCL | 0248_05.05 |
| 18 | W22-KRN1L_IN0833 RCL | 0833_29.04 |
| 19 | W22-KRN1L_IN1424 RCL | 1424_45.08 |
| 20 | W22-KRN1L_IN0736 RCL | 0736_25.15 |
| 21 | W22-KRN1L_IN0904 RCL | 0904_32.05 |
| 22 | W22-KRN1L_IN1337 RCL | 1337_42.08 |
| 23 | W22-KRN1L_IN0179 RCL | 0179_04.05 |
| 24 | W22-KRN1L_IN1811 RCL | 1811_54.02 |
| 25 | W22-KRN1L_IN1419 RCL | 1419_44.14 |
| 26 | W22-KRN1L_IN0935 MCL | 0935_37.02 |
| 27 | W22-KRN1L_IN1053 RCL | 1053_39.12 |
| 28 | W22-KRN1L_IN1453 RCL | 1453_46.19 |
| 29 | W22-KRN1L_IN1806 RCL | 1806_53.03 |
| 30 | W22-KRN1L_IN1113 RCL | 1113_40.01 |
| 31 | W22-KRN1L_IN0618 RCL | 0618_20.06 |
| 32 | W22-KRN1L_IN1806 MCL | 1806_53.08 |
| 33 | W22-KRN1L_IN1826 RCL | 1826_55.13 |
| 34 | W22-KRN1L_IN0051 RCL | 0051_02.06 |
| 35 | W22-KRN1L_IN0935 RCL | 0935_37.07 |
| 36 | W22-KRN1L_IN0427 RCL | 0427_11.04 |
| 37 | W22-KRN1L_IN0562 RCL | 0562_16.01 |
| 38 | W22-KRN1L_IN0502 RCL | 0502_14.01 |
| 39 | W22-KRN1L_IN0315 RCL | 0315_09.01 |
| 40 | W22-KRN1L_IN0433 RCL | 0433_12.03 |
| 41 | W22-KRN1L_IN1557 RCL | 1557_48.07 |
| 42 | W22-KRN1L_IN1785 RCL | 1785_52.03 |
| 43 | W22-KRN1L_IN0853 RCL | 0853_30.04 |
| 44 | W22-KRN1L_IN0736 TCL | 0736_25.25 |
| 45 | W22-KRN1L_IN0407 RCL | 0407_10.04 |
| 46 | W22-KRN1L_IN0611 RCL | 0611_18.10 |
| 47 | W22-KRN1L_IN0680 RCL | 0680_21.04 |
| 48 | W22-KRN1L_IN1424 TCL | 1424_45.05 |
| 49 | W22-KRN1L_IN0689 RCL | 0689_23.10 |
| 50 | W22-KRN1L_IN0050 RCL | 0050_01.01 |
| 51 | W22-KRN1L_IN0074 RCL | 0074_03.11 |
| 52 | W22-KRN1L_IN1053 TCL | 1053_39.13 |
| 53 | W22-KRN1L_IN0881 RCL | 0881_31.10 |
| 54 | W22-KRN1L_IN0179 TCL | 0179_04.04 |
| 55 | W22-KRN1L_IN1321 RCL | 1321_41.08 |
| 56 | W22-KRN1L_IN0433 TCL | 0433_12.01 |
| 57 | W22-KRN1L_IN1743 TCL | 1743_50.05 |
| 58 | W22-KRN1L_IN0279 RCL | 0279_08.04 |
| 59 | W22-KRN1L_IN0853 TCL | 0853_30.13 |
| 60 | W22-KRN1L_IN0792 RCL | 0792_26.09 |
| 61 | W22-KRN1L_IN0257 RCL | 0257_06.05 |
| 62 | W22-KRN1L_IN0553 RCL | 0553_15.09 |
| 63 | W22-KRN1L_IN0614 RCL | 0614_19.03 |
| 64 | W22-KRN1L_IN0142 TCL | 0142_34.01 |
| 65 | W22-KRN1L_IN0074 TCL | 0074_03.04 |
| 66 | W22-KRN1L_IN0918 TCL | 0918_36.03 |
| 67 | W22-KRN1L_IN0618 TCL | 0618_20.04 |
| 68 | W22-KRN1L_IN1360 RCL | 1360_43.06 |

Stock indicates the genomic background of the line, the chromosome number and arm size, the phenotypic trait the line was used to measure and the genotypic class: RCL, MCL or TCL. RCL, recombinant control line; MCL, maize control line; TCL, teosinte control line. Seed lot indicates Doebley lab seed catalog numbering system.
