# Supplementary material for: Fine Mapping of a QTL Associated with Kernel Row Number on Chromosome 1 of Maize
Source: PLoS One. 2016 Mar 1;11(3):e0150276. doi: 10.1371/journal.pone.0150276 (PMC4773258; doi:10.1371/journal.pone.0150276)
Supplement: S3 Table — (DOCX) [file pone.0150276.s004.docx]

**S3 Table. Expression Data of the Genes located within the 1.5 LOD Confidence Interval of KRN1.4**

| **No.** | **Gene models** | **B73 RefGen_v2** | **Functional characterization ^a^** | **RNAseq read counts in the immature ear ^b^** | | | | **p value** | | | **Regulatory** | **Crosses** |
| --- | --- | --- | --- | --- | --- | --- | --- | --- | --- | --- | --- | --- |
|  |  |  |  | **H.Maize** | **H.Teosinte** | **P.Maize** | **P.Teosinte** | **Hybrid** | **Parents** | **Trans** | **category** |  |
| 1 | AC206259.3_FGT003 | chr1:292,660,134-292,671,866 | Protein coding | 988 | 1041 | 579 | 732 | 0.25 | 0.00 | 0.01 | Trans only | 21 |
| 2 | GRMZM2G409839 | chr1:292,694,407-292,704,781 | Protein coding | 1203 | 1009 | 667 | 520 | 0.00 | 0.00 | 0.33 | Cis only | 27 |
| 3 | GRMZM2G405690 | chr1:292,705,562-292,709,078 | Protein coding | 106 | 86 | 68 | 91 | 0.17 | 0.08 | NA | Conserved | 2 |
| 4 | GRMZM2G073155 | chr1:292,796,431-292797030 | Protein coding | NA | NA | NA | NA | NA | NA | NA | NA | NA |
| 5 | AC225147.4_FG003 | chr1:292,875,307-292879433 | Alfin-like transcription factor 15; PHD finger protein | 483 | 521 | 318 | 236 | 0.24 | 0.00 | 0.00 | Trans only | 20 |
| 6 | AC225147.4_FG002 | chr1:292,879,723-292,882,219 | Ribosomal protein S23 family protein | 2134 | 1965 | 1748 | 1379 | 0.01 | 0.00 | 0.00 | Cis + Trans | 11 |
| 7 | GRMZM5G862109 | chr1:292,889,740-292893983 | *Indeterminate spikelet 1* (*ids1*), *tasselseed 6* (*ts6*) | 2588 | 2334 | 1037 | 1595 | 0.00 | 0.00 | 0.00 | Cis x Trans | 20 |

B73 RefGen_v2: B73 reference genome sequence assembly version 2. H.Maize refers to hybrid maize, H.Teosinte refers to hybrid teosinte, P.Maize refers to parent maize, P.Teosinte refers to parent teosinte. Trans indicates the *p* value for a test measuring the effect of trans expression in the gene. Crosses refers to the number of crosses that were done to obtain the expression data for each gene.

**^a^** Information retrieved from the Maize Genetics and Genomics Database, MaizeGDB [1, 2]

**^b^** Expression data based RNAseq analysis from Lemmon et al. [3]

1. Lawrence CJ, Dong Q, Polacco ML, Seigfried TE, Brendel V. MaizeGDB, the community database for maize genetics and genomics. Nucleic Acids Res. 2004;32(Database issue):D393-7. doi: 10.1093/nar/gkh011. PubMed PMID: 14681441; PubMed Central PMCID: PMCPMC308746.

2. Sen TZ, Harper LC, Schaeffer ML, Andorf CM, Seigfried TE, Campbell DA, et al. Choosing a genome browser for a Model Organism Database: surveying the maize community. Database (Oxford). 2010;2010:baq007. doi: 10.1093/database/baq007. PubMed PMID: 20627860; PubMed Central PMCID: PMCPMC2911842.

3. Lemmon ZH, Bukowski R, Sun Q, Doebley JF. The role of cis regulatory evolution in maize domestication. PLoS Genet. 2014;10(11):e1004745. doi: 10.1371/journal.pgen.1004745. PubMed PMID: 25375861; PubMed Central PMCID: PMCPMC4222645.
